# ﻿Molecular phylogeny and revision of species groups of Nearctic bombardier beetles (Carabidae, Brachininae, *Brachinus* ( *Neobrachinus*))

**DOI:** 10.3897/zookeys.1131.85218

**Published:** 2022-11-23

**Authors:** Raine M. Ikagawa, Wendy Moore

**Affiliations:** 1 Graduate Interdisciplinary Program in Entomology and Insect Science, University of Arizona, Tucson, Arizona, 85721-0036, USA University of Arizona Tucson United States of America; 2 Department of Entomology, University of Arizona, 1140 E. South Campus Dr., Tucson, Arizona, 85721-0036, USA University of Arizona Tucson United States of America

**Keywords:** molecular phylogenetics, systematics

## Abstract

Bombardier beetles of the genus *Brachinus* Weber are notorious for their explosive defensive chemistry. Despite ongoing research on their defense mechanism, life history, and ecology, the group lacks a robust molecular-based phylogeny. In this study, three loci from mitochondrial and nuclear genomes (COI, CAD, 28S) are used to reconstruct the phylogeny of the large subgenus Neobrachinus, and test species group boundaries hypothesized by Erwin (1970) based on morphological characters. Erwin’s *fumans* species group is found to be polyphyletic, and is herein re-defined with eight new species groups erected to reflect clades based on molecular evidence: the *cinctipennis*, *cyanipennis*, *galactoderus*, *gebhardis*, *mexicanus*, *phaeocerus*, *quadripennis*, and *tenuicollis* species groups. Erwin’s *cordicollis* group is also expanded to include Brachinus (Neobrachinus) medius and the *americanus* group.

## ﻿Introduction

Bombardier beetles of the genus *Brachinus* Weber are famous for their explosive defensive chemistry; when provoked, they generate a 100 °C cloud of benzoquinones and aim the explosion towards their enemy (Eisner 1958; Schildknecht et al. 1964; Aneshansley et al. 1969; Dean et al. 1990; Arndt et al. 2015). *Brachinus* are abundant predators and scavengers in their communities, they offer other carabids (e.g., certain species of *Agonum* Bonelli, *Chlaenius* Bonelli, and *Platynus* Bonelli) well-protected spaces in multispecies aggregations (Schaller et al. 2018), and they have the potential to sustainably manage pest populations in agroecosystems (Scaccini et al. 2020). Previous research has also examined their larval development (Erwin 1967; Juliano 1984; Juliano 1985; Saska and Honek 2004), aggregation behaviors (Brandmayr et al. 2006; Schaller et al. 2018), and microbiome (McManus et al. 2018; Silver et al. 2021).

**Figure 1.**
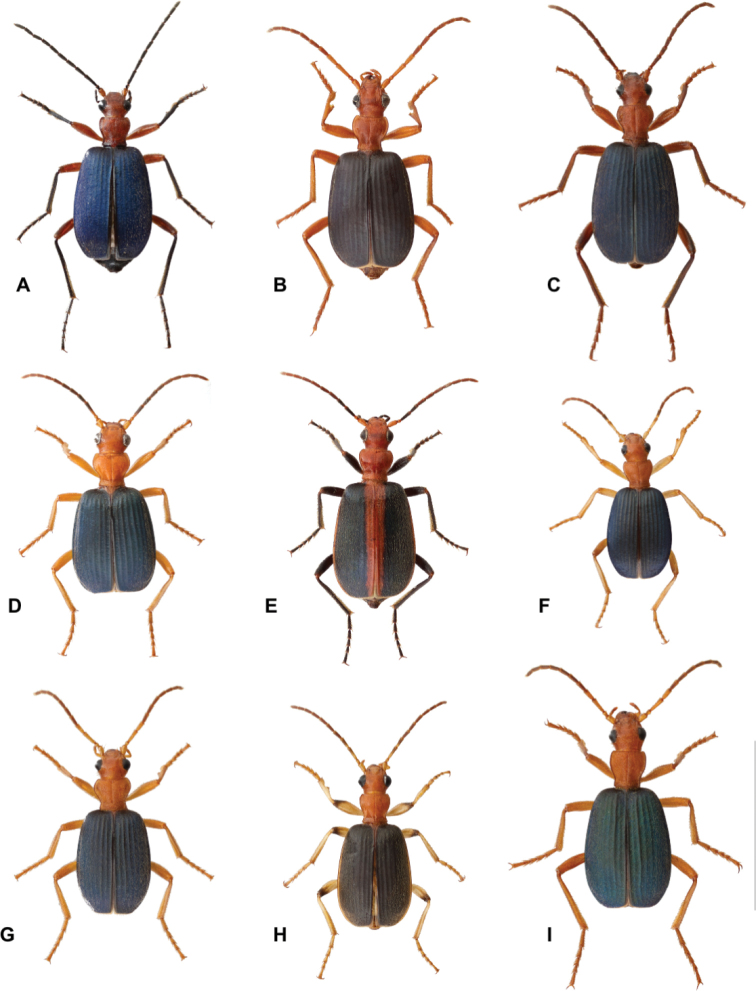
Dorsal habitus view of representatives from several species groups of the subgenus Neobrachinus Erwin **A***B.azureipennis* Chaudoir **B***B.gebhardis* Erwin **C***B.elongatulus* Chaudoir **D***B.mexicanus* Dejean **E***B.cibolensis* Erwin **F***B.costipennis* Motschulsky **G***B.hirsutus* Bates **H***B.lateralis* Dejean **I***B.favicollis* Erwin. Scale bar: 1 cm.

Species of the BrachinussubgenusNeobrachinus Erwin have historically been described as difficult to identify. George Ball (1960) wrote: “The taxonomy of the North American species of this group is very poorly understood and it is almost a waste of time at present to attempt to determine individuals to species.” Ten years later, Erwin (1970) revised the Nearctic members of the genus after studying more than 28,000 specimens of *Brachinus* and more than 2,000 specimens of other brachinine taxa. He identified many subtle species-specific characters, from the depth of punctures on the pronotum to the shape of the miniscule virga of the endophallus which requires meticulous dissection and processing to observe. He also used morphological characters to classify 62 members of *Neobrachinus* into 14 species groups (representatives in Fig. 1) and to propose a phylogenetic tree. He hypothesized that speciation among *Neobrachinus* was mirrored by the evolution of the shape of the virga, which is the apical sclerite surrounding the gonopore of the male endophallus. He placed the *americanus* group at the base of the tree based on morphological similarities of the virga with a species known from Sikkim, India, *B.dryas* Andrewes (Erwin 1970). The virga of *B.dryas* was regarded as a *Neobrachinus*-type, different from all other virgae of *Brachinus* species outside of the Americas (Erwin 1970); *B.dryas* has since been reclassified and placed in the subgenus Brachynolomus Reitter (Akhil et al. 2020).

The vast majority of species examined in Erwin (1970) are endemic to North and Central America. The subgenus also includes 21 *incertae sedis* species from South America that were not examined. Erwin hypothesized that the ancestral lineage of *Neobrachinus* entered North America via the Bering Land Bridge and rapidly invaded South America in a single colonization event before its isolation from Central America during the Eocene. He considered that these *incertae sedis* species were likely members of species groups *brunneus*, *grandis*, *lateralis*, and *texanus*, but stated that further examinations of South American taxa would be necessary before placing them in species groups.

**Figure 2.**
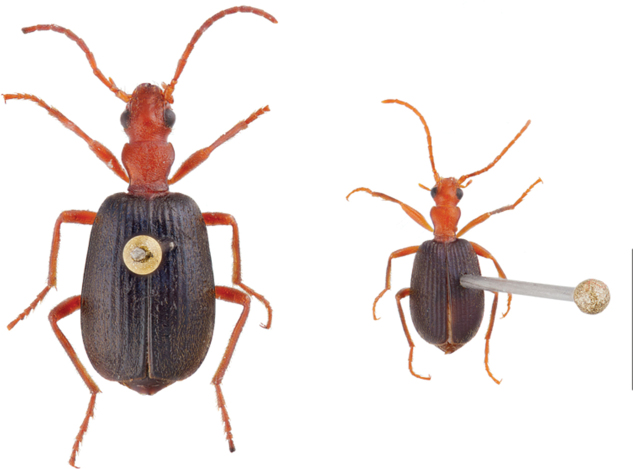
Two specimens of *B.elongatulus* demonstrating adult size variation within the species. Scale bar: 5 mm.

Erwin’s work transformed brachinine taxonomy and provided a dichotomous key for identifying brachinine genera and North and Central American *Neobrachinus* species. However, identification of *Neobrachinus* species remains challenging. This is largely due to highly conserved morphology; the maintenance of “the *Brachinus* habitus” seems to have been favored over the course of multiple speciation events (e.g., Fig. 1C, D, F, G, I). Furthermore, species-level identification often relies on very subtle characters that can change over in specimens time as colors darken and setae break. Adding to the challenge, members of the same species can vary significantly in size because of their idiobiont ectoparasitoid larval lifestyle (Fig. 2). Upon hatching, the first instar triungulin locates the pupa of an aquatic beetle and consumes it (and only it) during the course of its development. Therefore, adult size is positively correlated to the size of the pupal host, resulting in vast differences in adult body size (Juliano 1985; Saska and Honek 2012). Another barrier to identification is that some species of *Neobrachinus* are only represented by a few specimens collected many decades ago, deposited in a handful of museum collections. Not only does this hinder morphological work, with so few specimens available for comparison, it is also more challenging to acquire molecular sequence data, particularly from single-copy genes.

*Neobrachinus* are abundant members of riparian arthropod communities in the southwestern US (Moody and Sabo 2017). A recent study documenting and exploring the multispecies aggregation behavior of these species included a molecular phylogeny of many *Neobrachinus* species (Schaller et al. 2018). This work corroborated the monophyly of *Neobrachinus* using molecular sequence data. However, results also suggested that some species groups within *Neobrachinus* may not be monophyletic, including the *fumans* group, and therefore some diagnostic morphological characters identified by Erwin (1970) may be plesiomorphic. For example, Erwin considered the shape of the virga “with sides curved over ventrally from base to apex, forming a central (ventral) trough” to be the apomorphy of the *fumans* species group (Erwin 1970).

With morphologically challenging taxa, molecular sequence data are often used to determine species boundaries and relationships, and these studies also help to reveal cryptic diversity. This study aims to address the morphological challenges of *Neobrachinus* by using molecular sequence data to infer the phylogeny of the species and to test proposed species groups.

## ﻿Materials and methods

### ﻿Taxon sampling and classification

Challenges associated with *Neobrachinus* identification led us to limit our taxon sampling to expertly identified specimens in museum collections (Suppl. material 1: table S1). We targeted material from institutions where Erwin conducted work on *Neobrachinus*. Specimen loans were acquired from the University of Alberta E.H. Strickland Entomological Museum (Edmonton, Alberta, CA), where Erwin deposited his vouchers after completing his PhD research with George E. Ball, culminating in several publications (Erwin 1965, 1967, 1970, 1973). We also used specimens deposited in the University of Arizona Insect Collection (Tucson, Arizona, USA), where Erwin identified *Brachinus* specimens to the species level as a Visiting Arthropod Systematist in 2014.

Efforts were made to sample several species from as many species groups as possible, especially within the large *fumans* group which we hypothesized may not be monophyletic. We also downloaded all available sequences of *Neobrachinus* species from the Barcode of Life Database (BOLD) and GenBank and tested their species identities against sequence data from expertly identified specimens.

### ﻿DNA extraction and quantification

Total genomic DNA was extracted from the right middle leg of specimens using the Qiagen DNeasy Blood & Tissue Kit (Valencia, CA) following the manufacturer suggested protocol. Extractions on older specimens were conducted in the Schlinger Ancient DNA Laboratory at the University of Arizona Insect Collection using the QIAamp DNA Micro Kit (Qiagen Inc., Valencia, CA) following the manufacturer suggested protocol. The concentration of total genomic DNA in extraction products was measured on a Qubit 3.0 Fluorometer (Thermo Fisher, USA). Samples with quantifiable DNA were used in subsequent PCRs.

### ﻿Gene selection and PCR

The gene regions CAD (carbamoyl-phosphate synthetase 2, aspartate transcarbamylase, dihydroorotase) and COI (cytochrome c oxidase subunit I) have been shown to be phylogenetically informative in *Neobrachinus* by Schaller et al. (2018), and sequences generated in that study were downloaded from GenBank. Sequences for additional taxa to these datasets were generated following published protocols (Schaller et al. 2018).

Sequence data were also obtained for the D2-3 region of large subunit ribosomal gene (28S) from the total genomic DNA extracted for Schaller et al. (2018) as well as the new taxa added herein. We chose to add 28S to our analyses for several reasons; it has been shown to be phylogenetically informative in other genera of carabid beetles, and as a multicopy gene it is easier to amplify from older museum specimens (Sproul and Maddison 2017). Therefore, we started building a reference library of 28S sequences obtained from expertly identified specimens to facilitate future molecular work with museum material. The D2-3 region of 28S was amplified using primers LS58F and LS998R (Ober 2002) and the following PCR cycling conditions: initial denaturation at 94 °C for 2 min, followed by 35 cycles at 94 °C for 22 s, 57 °C for 22 s, 72 °C for 1 min and 10 s, and a final elongation at 72 °C for 5 min.

### ﻿Sequencing

PCR products were quantified, normalized, and sequenced in forward and reverse directions using Sanger sequencing at the University of Arizona Genetics Core (UAGC) using an Applied Biosystems 3730 DNA Analyzer (ThermoFisher Scientific). Chromatograms were assembled into contigs, and initial base calls were made using Phred (Green and Ewing 2002) and Phrap (Green 1999) as implemented by the Chromaseq 1.52 module (Maddison and Maddison 2020) within Mesquite 3.61 (Maddison and Maddison 2019). Final base calls were made by visual inspection of the contigs.

### ﻿Phylogenetic analysis

Three single gene matrices (COI, CAD, and 28S) were assembled. Each matrix contained sequences generated specifically for this study as well as all homologous sequences of *Neobrachinus* publicly available on BOLD and GenBank (databases searched January 2021) (Suppl. material 1: table S1). The COI matrix contained 270 taxa, the CAD matrix contained 70 taxa, and the 28S matrix contained 54 taxa. Sequences in each matrix were aligned using default settings in MAFFT v. 7.474 (Katoh and Standley 2013) within Mesquite. A concatenated matrix with the data from all three genes was also assembled, which contained 282 taxa including 228 *Neobrachinus* (representing 9/15 *Neobrachinus* species groups, and 32/62 *Neobrachinus* species) and 54 outgroups. In the concatenated matrix and single gene matrices, COI and CAD characters were partitioned by codon position. Maximum-likelihood analyses and bootstrap analyses were conducted on single gene matrices and on the concatenated dataset using IQ-TREE v. 1.6.10 (Nguyen et al. 2015). The ModelFinder feature within IQ-TREE (Kalyaanamoorthy et al. 2017) was used to find the optimal character evolution models. The ModelFinder Plus model option was used for 28S, and the TESTMERGE option for the protein-coding genes and for the concatenated dataset. One hundred searches were conducted for the maximum-likelihood tree for each matrix in Mesquite. Bootstrap analyses for the four trees were conducted with 1000 replicates using IQ-TREE v. 1.6.10 (Nguyen et al. 2015), as orchestrated by the CIPRES Science Gateway (Miller et al. 2010). Support for and against clades were calculated for each species group and the subgenus Neobrachinus in Mesquite using its “Clade Frequencies in Trees” feature and bootstrap trees generated by IQ-TREE (Maddison et al. 2019; Kavanaugh et al. 2021).

## ﻿Results

### ﻿Models and partitions

IQ-Tree ModelFinder and ModelFinder Plus identified the following models of evolution for each character partition: COI codon 1 = K2P+I+G4; COI codon position 2, CAD codon 1, CAD codon 2 = 2K2P+I; COI codon 3 = TIM2+F+G4; CAD codon 3 = HKY+F; and 28S = GTR+F+I+G4.

### ﻿Molecular phylogeny

**Figure 3.**
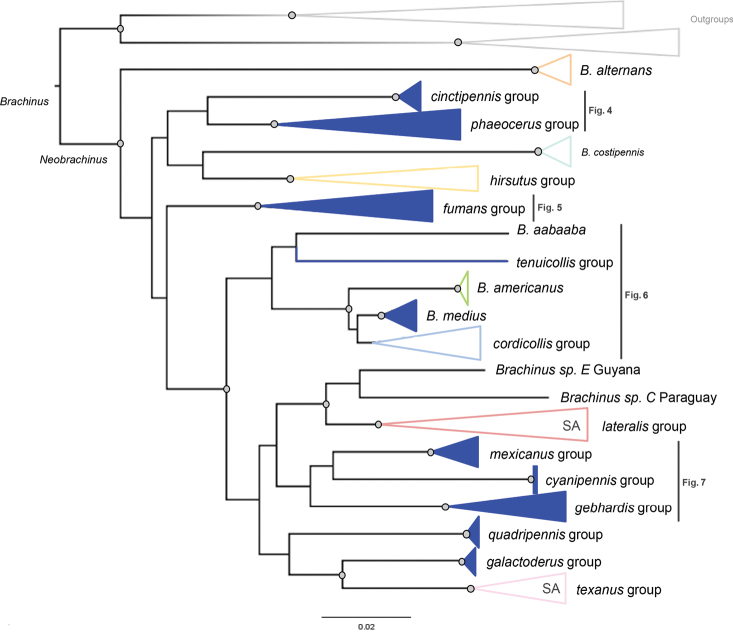
The maximum-likelihood three-gene molecular phylogeny of *Neobrachinus* with clades collapsed. Clades are colored by species group. Clades in solid blue were formerly placed within the *fumans* species group. Nodes with bootstrap values > 0.90 are denoted with grey circles. Clades present in South America are denoted with “SA.”

**Figure 4.**
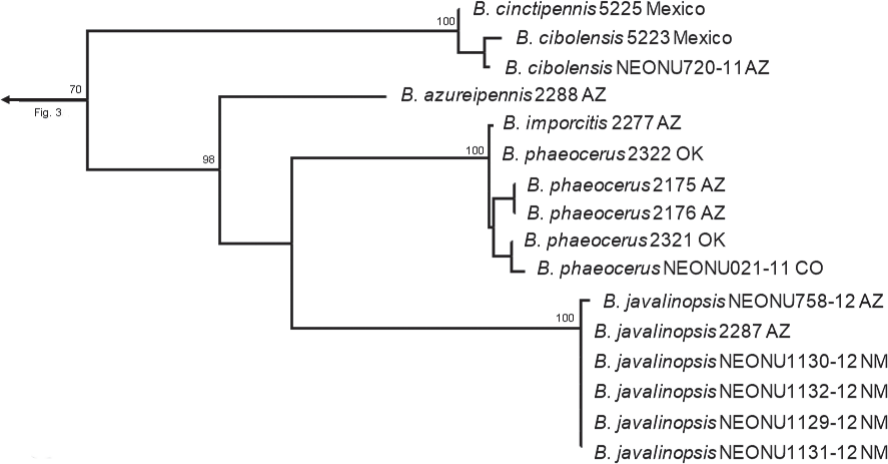
Phylogeny within the *cinctipennis* and *phaeocerus* species groups.

**Figure 5.**
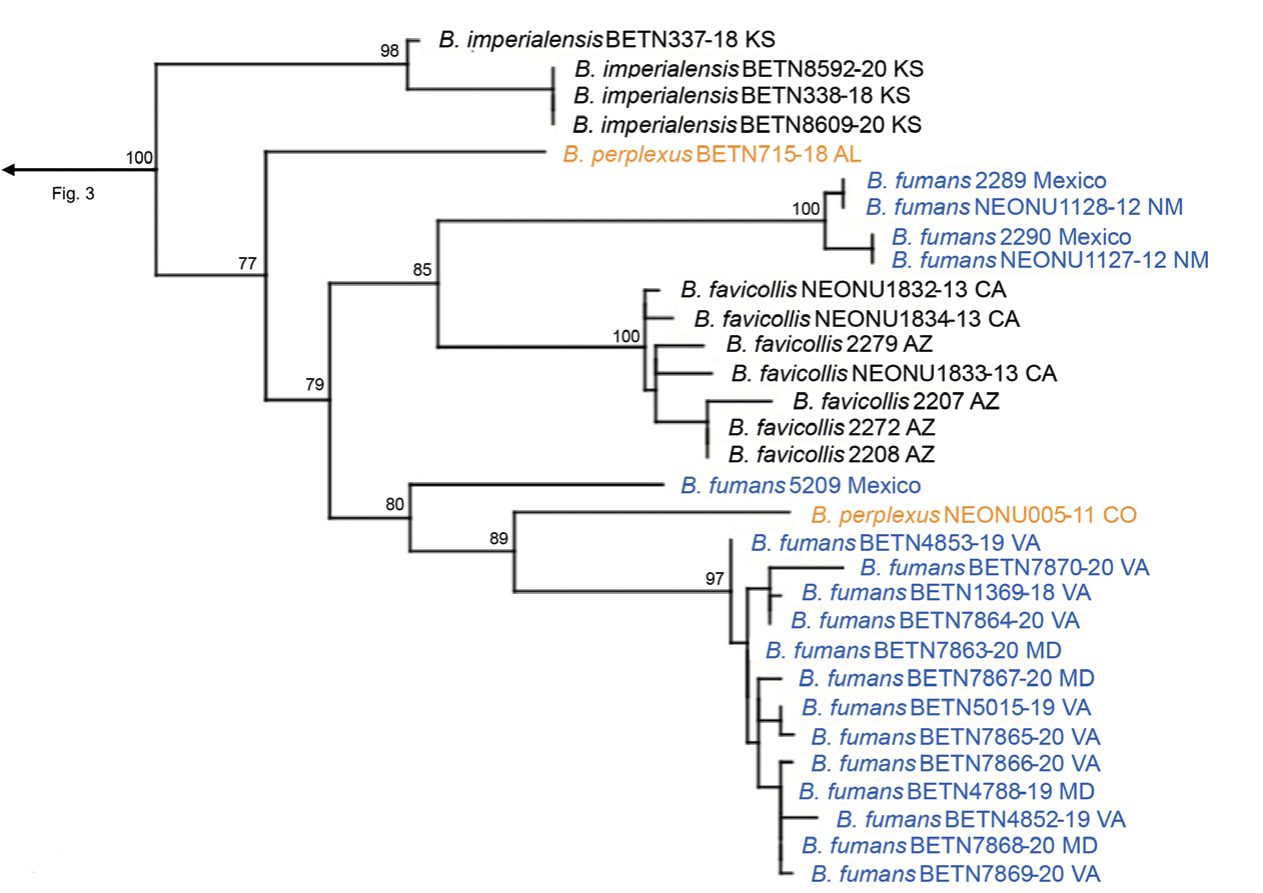
Phylogeny within the *fumans* species group, *B.fumans* colored blue and *B.perplexus* colored orange to highlight cryptic diversity and/or potential misidentifications of sequences on public databases.

**Figure 6.**
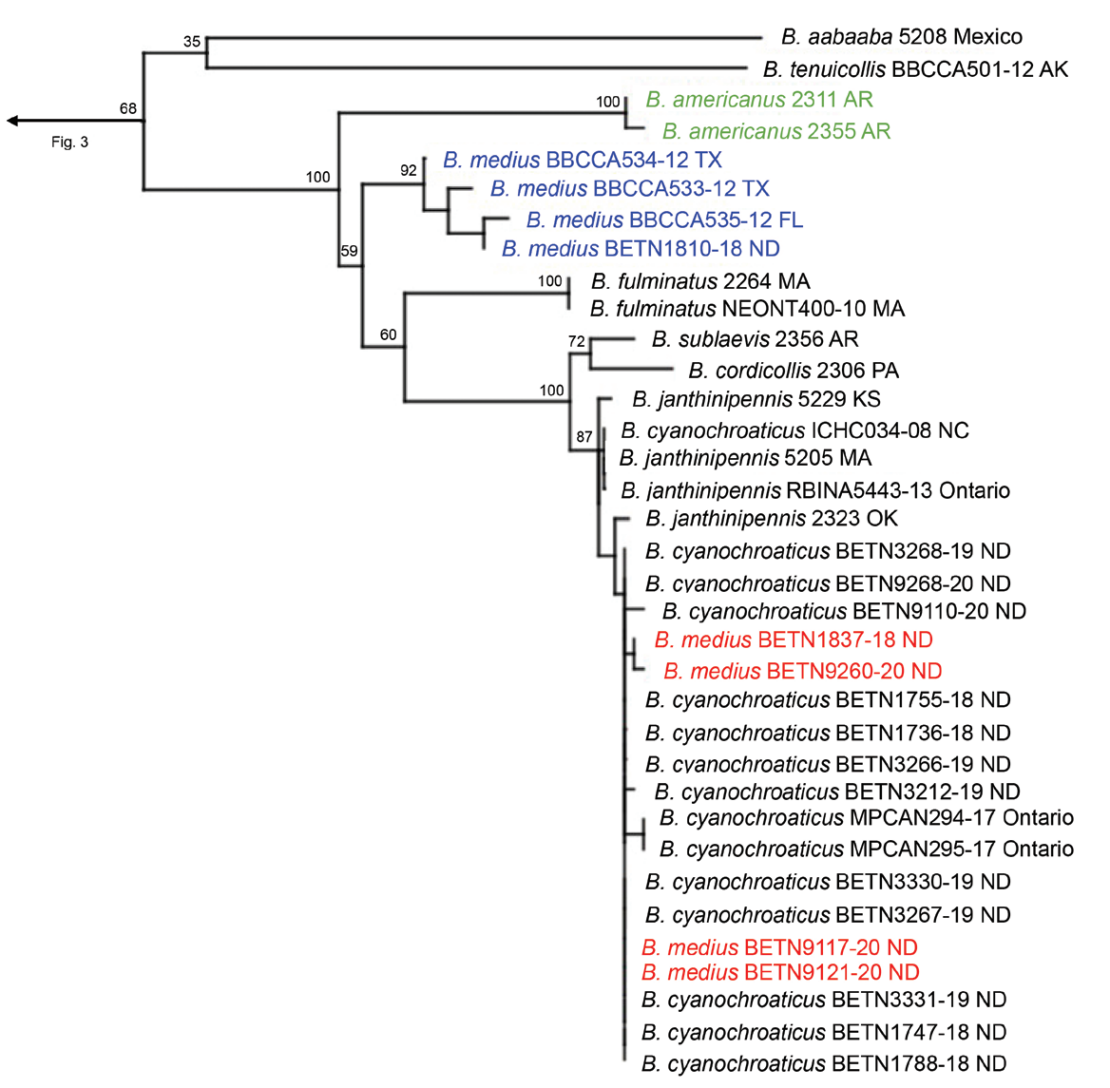
Phylogeny within the *tenuicollis*, *americanus*, *medius*, and *cordicollis* species groups. Novel additions to the *cordicollis* group are color-coded: *americanus* group (green) and *B.medius* (blue). Misidentifications of sequences on public databases are colored red.

**Figure 7.**
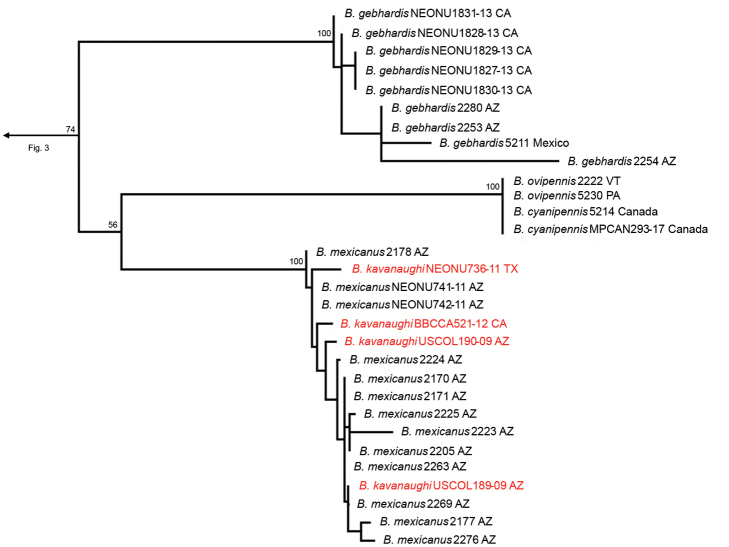
Phylogeny within the *mexicanus*, *cyanipennis*, and *gebhardis* species groups. *B.kavanaughi* colored red to highlight potential misidentifications of sequences or the need to synonymize this species with *B.mexicanus*.

The three-gene IQ-Tree analysis resulted in the phylogeny shown in Figs 3–7. The full concatenated tree, and individual gene trees for 28S, CAD, and COI, are shown in Suppl. material 1: figs S1–S4). The COI analysis identified several specimens obtained from public databases that could be misidentified (Suppl. material 1: fig. S4). In all analyses, the following species groups originally proposed by Erwin 1970 were recovered as monophyletic: *cordicollis*, *lateralis*, and *texanus*.

**Figure 8.**
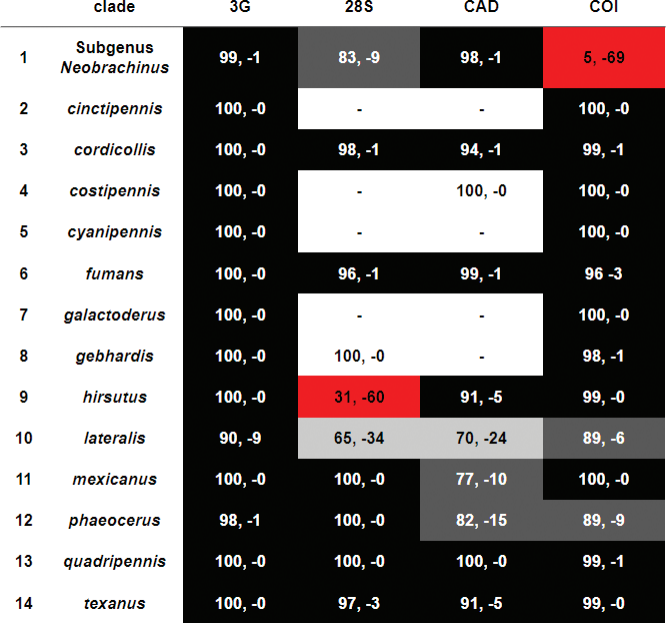
Bootstrap support for and against clades of *Neobrachinus*. Each column has maximum likelihood bootstrap values as percentages for or against each clade recovered in each dataset: the three-gene concatenated matrix (3G), and the single-gene datasets, 28S, CAD, and COI. Positive values indicate support while negative numbers indicate support for the contradictory clade with the highest support. Cells with bootstrap values ≥ 90 are in black, values between 75 and 89 in dark grey, and values between 50 and 74 in light grey. Cells in red have bootstrap values for the contradictory clade ≥ 50.

In all analyses the *fumans* species group proposed by Erwin (1970) was polyphyletic (Fig. 3, Suppl. material 1: figs S1–S4). *B.medius* fell within a highly supported clade containing Erwin’s *americanus* and *cordicollis* groups (Fig. 6, Table 1). Support for and against the subgenus Neobrachinus and each species group are shown in Fig. 8. A revised classification of *Neobrachinus* species that reflects molecular and morphological support for species groups is shown in Table 1.

**Table 1.** Revised classification of Nearctic *Brachinus*. New species groups indicated with a triangle. Species groups and species not present in molecular phylogeny are indicated with an asterisk. Species present in South America are indicated with (SA). *Incertae sedis* taxa not considered in Erwin (1970) are indicated with a circle.

***aabaaba* species group**	***fumans* species group**△	***incertae sedis***
*B.aabaaba* Erwin	*B.fumans* (Fabricius)	*B.conformis* Dejean*
*B.sonorous* Erwin*	*B.favicollis* Erwin	*B.cyanipennis* Say
***alternans* species group**	*B.imperialensis* Erwin	*B.gebhardis* Erwin
*B.alternans* Dejean	*B.perplexus* Dejean	*B.kavanaughi* Erwin
*B.rugipennis* Chaudoir*	*B.puberulus* Chaudoir*	*B.mexicanus* Dejean
*B.viridipennis* Dejean*	*B.velutinus* Erwin*	*B.neglectus* LeConte
*brunneus* species group*	***galactoderus* species group**△	*B.oaxacensis* Erwin*
*B.brunneus* Laporte*	*B.galactoderus* Erwin	*B.ovipennis* LeConte
*B.melanarthrus* Chaudoir*	*grandis* species group*	*B.patruelis* LeConte*
***cinctipennis* species group**△	*B.grandis* Brullé ^SA^	*B.quadripennis* Dejean
*B.cinctipennis* Chevrolat	***hirsutus* species group**	*B.tenuicollis* LeConte
*B.cibolensis* Erwin	*B.hirsutus* Bates	*Brachinus sp. C* ^SA^
***cordicollis* species group**	*B.pallidus* Erwin	*Brachinus sp. E* ^SA^
*B.cordicollis* Dejean	*kansanus* species group*	*B.atramentarius* Mannerheim○^SA^*
*B.americanus* (LeConte)	*B.kansanus* LeConte*	*B.bilineatus* Laporte○^SA^*
*B.alexiguus* Erwin*	***lateralis* species group**	*B.bruchi* Liebke○^SA^*
*B.capnicus* Erwin*	*B.lateralis* Dejean	*B.fulvipennis* Chaudoir○^SA^*
*B.cyanochroaticus* Erwin	*B.adustipennis* Erwin	*B.fuscicornis* Dejean○^SA^*
*B.fulminatus* Erwin	*B.aeger* Chaudoir ^SA^	*B.genicularis* Mannerheim○^SA^*
*B.ichabodopsis* Erwin*	*B.arboreus* Chevrolat* ^SA^	*B.hylaenus* Reichardt○^SA^*
*B.janthinipennis* (Dejean)	*B.bilineatus* Castelnau*	*B.immarginatus* Brullé○^SA^*
*B.medius* T.W. Harris	*B.chalchihuitlicue* Erwin*	*B.intermedius* Brullé○^SA^*
*B.microamericanus* Erwin*	*B.chirriador* Erwin*	*B.limbiger* Chaudoir○^SA^*
*B.mobilis* Erwin	***phaeocerus* species group**△	*B.marginellus* Dejean○^SA^*
*B.oxygonus* Chaudoir*	*B.phaeocerus* Chaudoir	*B.marginiventris* Brullé○^SA^*
*B.sublaevis* Chaudoir	*B.azureipennis* Chaudoir	*B.niger* Chaudoir○^SA^*
*B.vulcanoides* Erwin*	*B.consanguineus* Chaudoir*	*B.nigricans* Chaudoir○^SA^*
***costipennis* species group**	*B.imporcitis* Erwin	*B.nigripes* G.R. Waterhouse○^SA^*
*B.costipennis* Motschulsky	*B.javalinopsis* Erwin	*B.olidus* Reiche○^SA^*
*explosus* species group*	***texanus* species group**	*B.pachygaster* Perty○^SA^*
*B.explosus* Erwin*	*B.texanus* Chaudoir*	*B.pallipes* Dejean○^SA^*
	*B.elongatulus* Chaudoir	*B.vicinus* Dejean○^SA^*
	*B.geniculatus* Dejean^SA^	*B.xanthophryus* Chaudoir○^SA^*
	*sallei* species group*	*B.xanthopleurus* Chaudoir○^SA^*
	*B.sallei* Chaudoir*	

## ﻿Discussion

### ﻿*Neobrachinus* species groups

This study used molecular data to test previous hypotheses of species group membership and phylogenetic relationships in the subgenus Neobrachinus that were proposed based on morphological data (Erwin 1970). The majority of Erwin’s species groups were recovered as monophyletic and were supported with high bootstrap values in all analyses (Figs 3–7). However, both molecular and morphological evidence support splitting the *fumans* species group into new species groups. Furthermore, relationships between species groups, for the most part, remain unclear.

The shape of the virga was not found to be phylogenetically informative as envisioned by Erwin (1970). For example, the revised *cordicollis* group now encompasses members of the former *americanus* group, as well as *B.medius* which was previously placed in the *fumans* group (Fig. 5). The virga of the *americanus* group was considered plesiomorphic among *Neobrachinus*, while the “H-shaped” virga of the *cordicollis* group was considered highly derived. Erwin (1970) also hypothesized that speciation within the subgenus Neobrachinus was largely connected to the evolution of the virga. Although molecular data largely corroborated species groups that were diagnosed by morphological characters, including the form of the virga, the polyphyly of Erwin’s *fumans* group indicates that molecular data is necessary to confirm the monophyly of species groups within *Neobrachinus*.

### ﻿Polyphyly of the *fumans* species group

Erwin’s *fumans* group contained 26 morphologically diverse species and was defined by a troughed virga. Considering the molecular evidence that supports splitting the *fumans* group, the troughed virga could be an ancestral or convergent form among *Neobrachinus*.

Species subgroups of the *fumans* group were also polyphyletic in the molecular phylogeny, highlighting potential convergent character states of the male genitalia. Members of Erwin’s *quadripennis* subgroup of the *fumans* group were recovered throughout the *Neobrachinus* tree: in the *phaeocerus* species group (Fig. 4), the *quadripennis* species group (Fig. 3), and the *mexicanus* species group (Fig. 7). This group was characterized by a ridge on the ventral surface of the male genitalia. The two members of Erwin’s *gebhardis* subgroup, *B.gebhardis* and *B.galactoderus*, were recovered in separate clades (Figs 3, 7); this group was characterized by the form of the median lobe of the male genitalia and the restriction of elytral pubescence to outer edge in the eighth depression.

Erwin’s *fumans* species group also contained seven monotypic species subgroups, of which four were included in this study. Two of these, *B.cyanipennis* and *B.ovipennis*, formed a clade and are now placed together in the *cyanipennis* species group (Fig. 7). As previously mentioned, *B.medius* had strong molecular support for its new placement in the revised *cordicollis* species group. Finally, *B.tenuicollis* remains a monotypic species group, as proposed by Erwin, with the support of molecular and morphological data (Fig. 6). All species of the *fumans* group that were in monotypic species groups not included in the molecular study, and all taxa not included in Erwin (1970) are considered *incertae sedis* (Table 1).

### ﻿Biogeographic implications

Erwin postulated all 84 species of *Neobrachinus* evolved from a single most recent common ancestor that crossed the Bering Land Bridge. The molecular phylogeny supports a Nearctic origin of the *Neobrachinus*, as predicted by Erwin (1970). Two clades, the *lateralis* and *texanus* species groups, have members that are present in South America (Figs 3–7). All other clades of *Neobrachinus* are only present in the Nearctic (Figs 3–7). He also hypothesized that the South American *Neobrachinus* species diversified from a single colonization event by an ancestral *Neobrachinus* lineage, giving rise to a monophyletic group containing the *brunneus*, *grandis*, *lateralis*, and *texanus* species groups (1970). However, the molecular phylogeny inferred in this study indicates otherwise. The two clades represented in this study with membership in South America, the *lateralis* and *texanus* species groups, are not sister taxa (Figs 3–7), indicating that multiple colonization events to South America must have occurred. Inclusion of additional South American taxa in a molecular phylogeny (Table 1) would illuminate their biogeographic history.

### ﻿Species identifications

Among the previously published sequences downloaded from public databases, molecular phylogenetic analysis revealed several cases where specimens were likely misidentified. Some specimens from North Dakota were identified as *B.medius* (BETN1837-18, BETN9260-20, BETN9117-20, BETN9121-20), however the sequences were in a well-supported clade, separate from *B.medius* from the same region (Fig. 6, Suppl. material 1: fig. S1).

Other potential misidentifications exist yet are difficult to confirm. For example, within the new *fumans* species group, there are several clades of the species *B.fumans* and *B.perplexus* (Fig. 5). Given the lack of molecular sequence data from other members of the species group, it is impossible to determine whether these clades represent cryptic diversity or whether the specimens are misidentified members of other species of the *fumans* group.

Another example is the clade containing *B.kavanaughi* and *B.mexicanus* (Fig. 7). Representatives of the species *B.kavanaughi* do not form a separate clade from *B.mexicanus*. Without examining the voucher specimens it is impossible to determine whether these specimens represent misidentified members of *B.mexicanus*, or whether *B.kavanaughi* should be synonymized with *B.mexicanus*.

## ﻿Conclusions

This research presents a molecular test of Erwin’s (1970) morphology-based hypothesis of *Neobrachinus* phylogeny, and our analyses largely support the monophyly of species groups posited in his enormous study. Utilizing multiple approaches and datasets for phylogenetic inference illuminates the power of integrative methods. Our finding that Erwin’s *fumans* species group was polyphyletic highlights the benefit of using molecular sequence data to infer phylogeny, especially in taxonomically and morphologically difficult groups like *Neobrachinus*.

Considering the challenges of morphological identification to the species level among *Neobrachinus*, molecular sequence data offer an accurate, alternative path to identification. Continued contribution of sequences from expertly identified specimens to libraries within databases such as BOLD and GenBank, will facilitate rapid, accessible, and accurate species identification. As sequencing technologies become cheaper and more readily available, acquiring sequence data for comparison in such databases is increasingly cost- and time-effective.

The present study elucidates the species group classification of more than half of the species of *Neobrachinus* detailed in Erwin (1970). We were able to place some species into molecularly defined species groups based on the presence of apomorphic morphological characters largely codified by Terry Erwin during the past 55 years (Erwin 1965, 1967, 1970). Many other species remain *incertae sedis*. Of those, most species are rarely collected and are known from few specimens collected long ago. Targeted efforts to acquire fresh material for molecular phylogenetic analysis, particularly of rare species, and the 22 species known only from Central and South America, will help provide a clearer picture of the evolutionary and biogeographic histories within *Neobrachinus*.

This systematic study of *Neobrachinus* emphasizes the importance of continued taxonomic and phylogenetic work to better understand their species boundaries, biogeography, and evolutionary history, and will enable future efforts to better understand these remarkable beetles.

## Supplementary materials

Supplementary material 1 Supplementary data Data type occurences, morphological, phylogenetic Explanation note IQ-Tree maximum-likelihood phylogeny on concatenated dataset containing 282 specimens of *Neobrachinus* and outgroups with bootstrap values. IQ-Tree maximum-likelihood phylogeny on 28S dataset containing 54 specimens of *Neobrachinus* and outgroups with bootstrap values. IQ-Tree maximum-likelihood phylogeny on CAD dataset containing 70 specimens of *Neobrachinus* and outgroups with bootstrap values. IQ-Tree maximum-likelihood phylogeny on COI dataset containing 270 specimens of *Neobrachinus* and outgroups with bootstrap values. Voucher specimens. Voucher number, collection information, and GenBank or BOLD accession numbers are provided for each specimen. https://binary.pensoft.net/file/773338This dataset is made available under the Open Database License (http://opendatacommons.org/licenses/odbl/1.0/). The Open Database License (ODbL) is a license agreement intended to allow users to freely share, modify, and use this Dataset while maintaining this same freedom for others, provided that the original source and author(s) are credited.Raine M. Ikagawa, Wendy Moore
