# Supplementary material for: ﻿Molecular phylogeny and revision of species groups of Nearctic bombardier beetles (Carabidae, Brachininae, Brachinus ( Neobrachinus))
Source: Zookeys. 2022 Nov 23;1131:155–71. doi: 10.3897/zookeys.1131.85218 (PMC9836565; doi:10.3897/zookeys.1131.85218)
Supplement: Supplementary material 1 — Supplementary data [file zookeys-1131-155_article-85218__-s001.pdf]

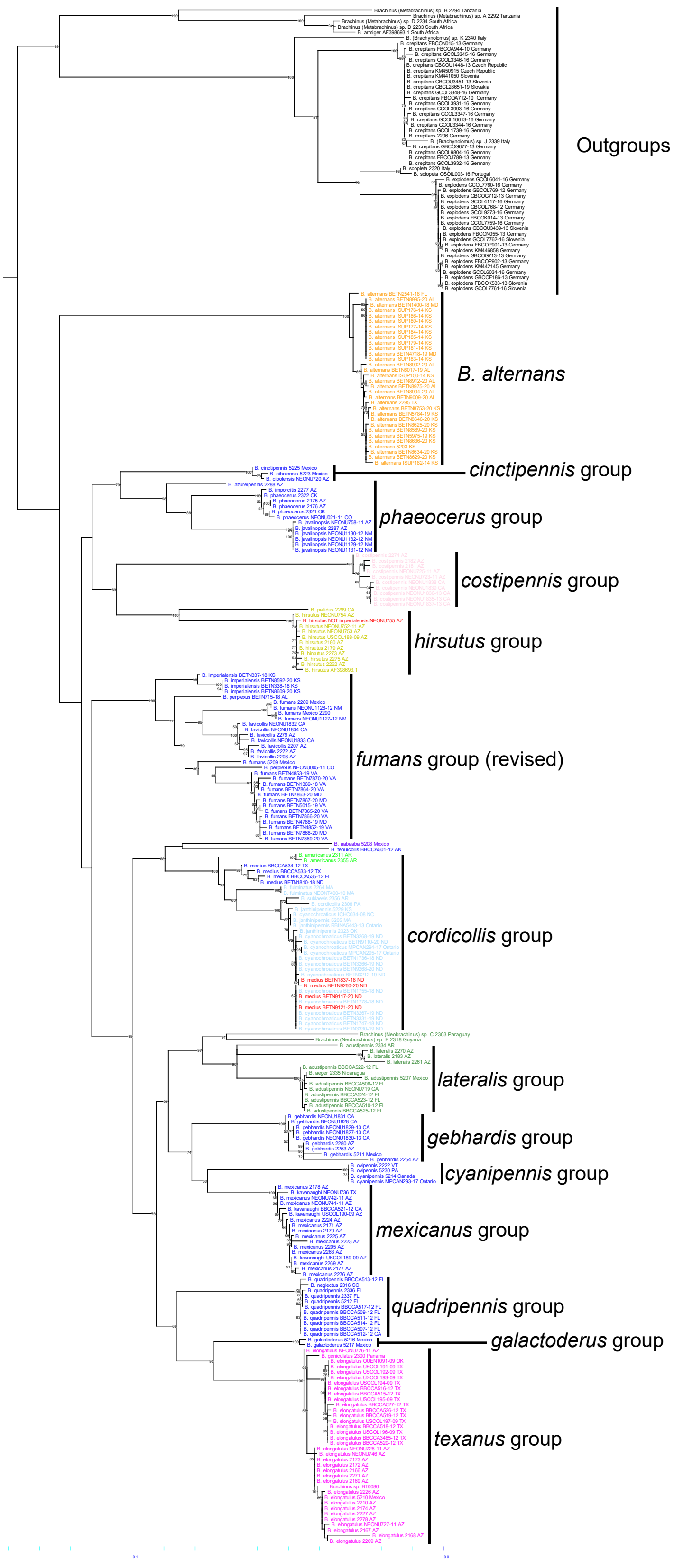

Figure S1. IQ-Tree maximum-likelihood phylogeny on concatenated dataset containing 282 specimens of *Neobrachinus* and outgroups with bootstrap values. Phylogeny is color-coded by species group. Royal blue = polyphyletic *fumans* group. Red = misidentified specimens.

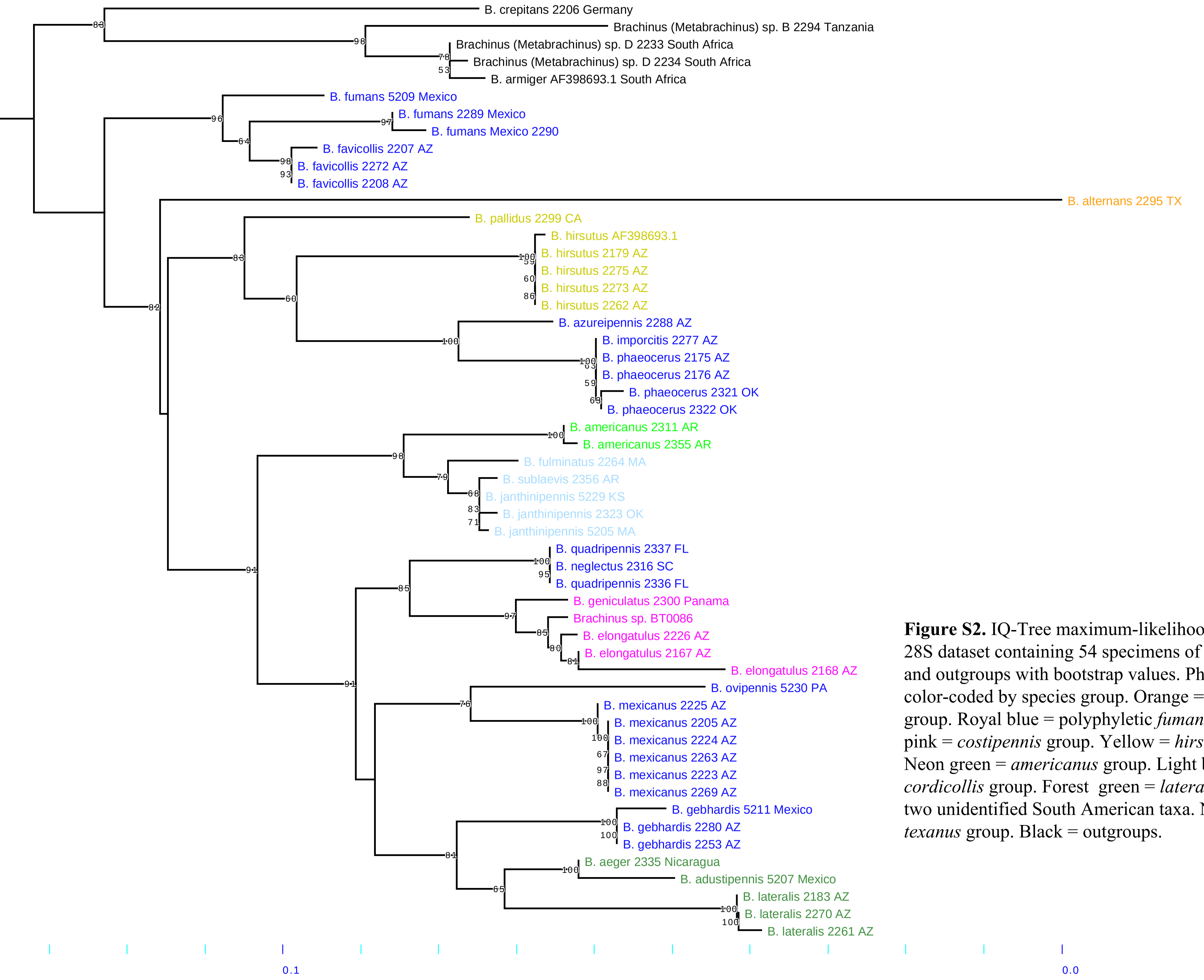

Figure S2. IQ-Tree maximum-likelihood phylogeny on 28S dataset containing 54 specimens of *Neobrachinus* and outgroups with bootstrap values. Phylogeny is color-coded by species group. Orange = *alternans* group. Royal blue = polyphyletic *fumans* group. Light pink = *costipennis* group. Yellow = *hirsutus* group. Neon green = *americanus* group. Light blue = *cordicollis* group. Forest green = *lateralis* group and two unidentified South American taxa. Neon pink = *texanus* group. Black = outgroups.

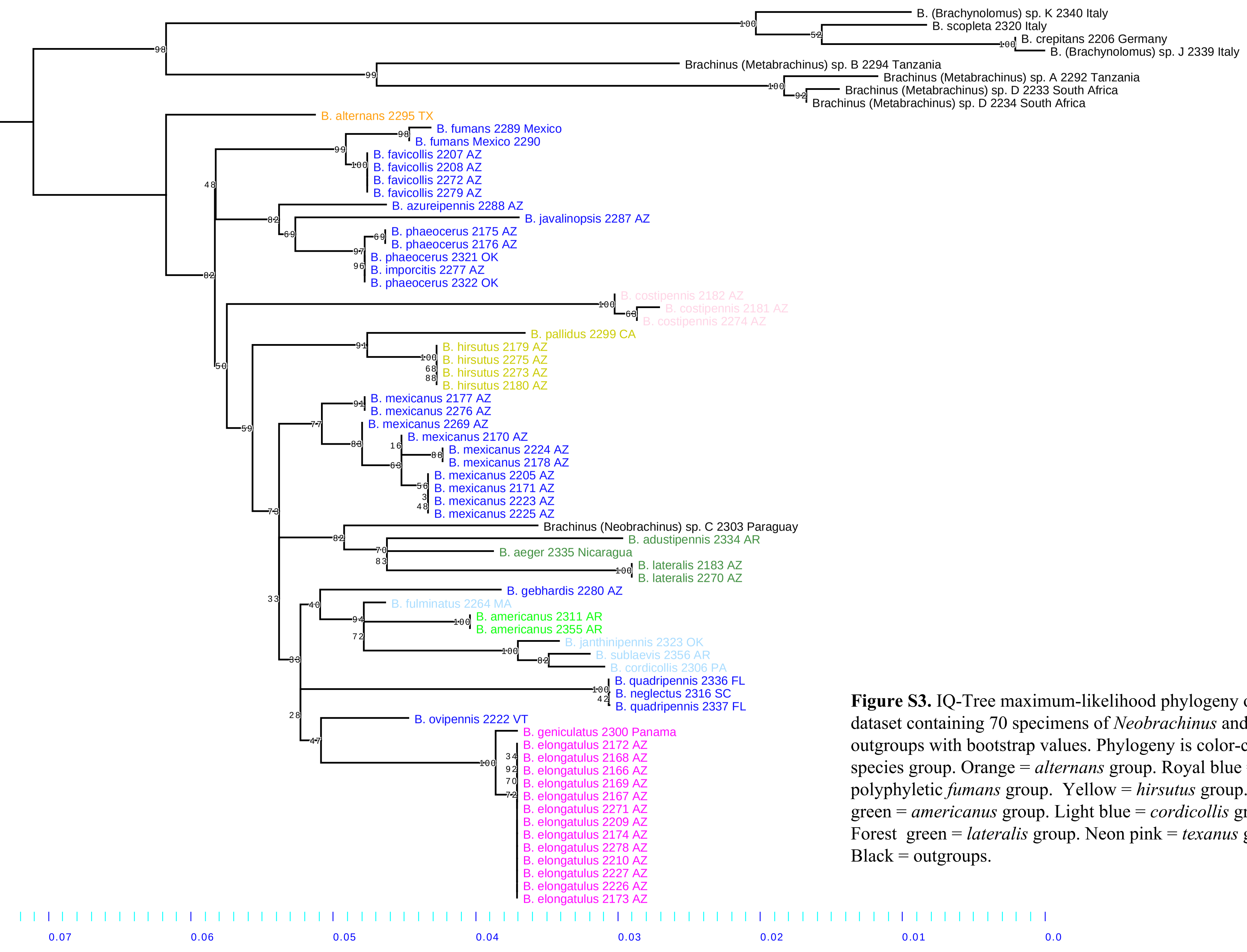

Figure S3. IQ-Tree maximum-likelihood phylogeny on CAD dataset containing 70 specimens of *Neobrachinus* and outgroups with bootstrap values. Phylogeny is color-coded by species group. Orange = *alternans* group. Royal blue = polyphyletic *fumans* group. Yellow = *hirsutus* group. Neon green = *americanus* group. Light blue = *cordicollis* group. Forest green = *lateralis* group. Neon pink = *texanus* group. Black = outgroups.

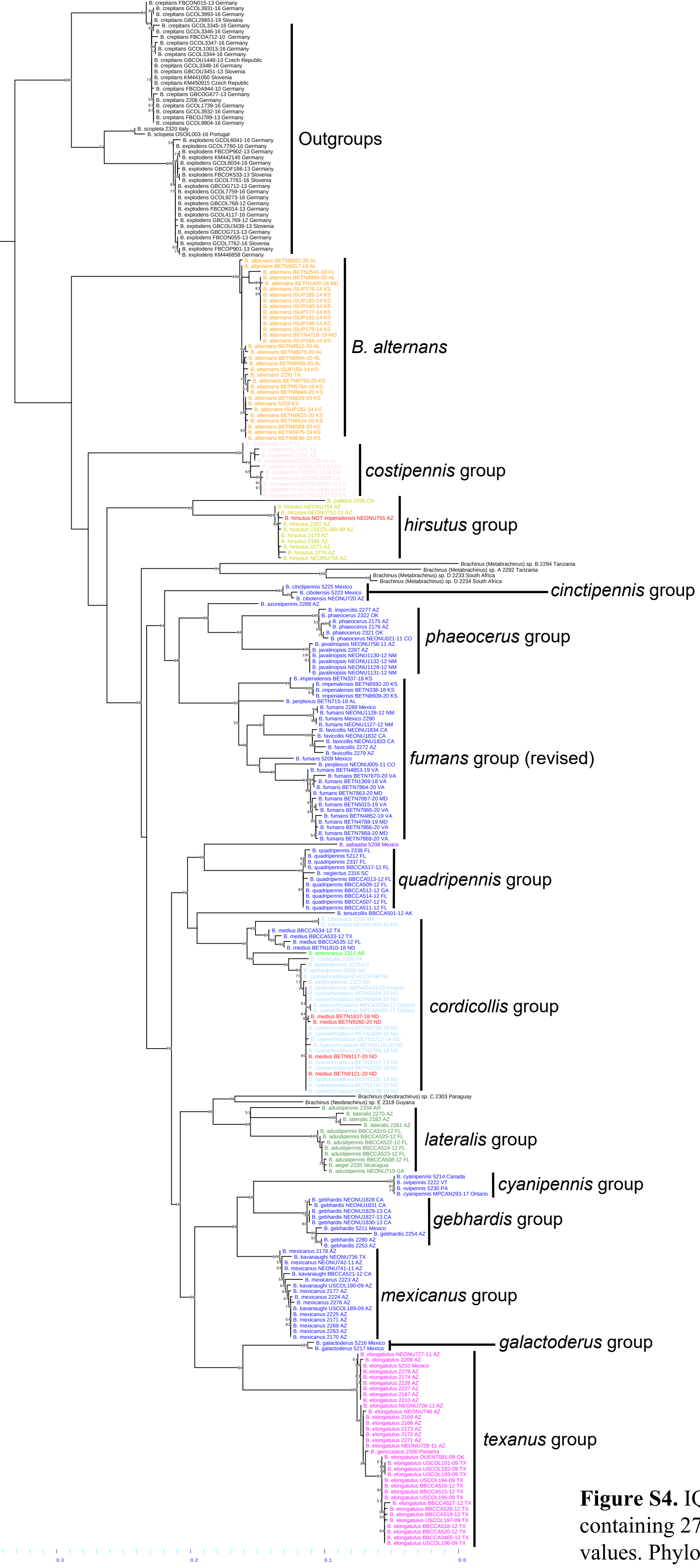

Figure S4. IQ-Tree maximum-likelihood phylogeny on COI dataset containing 270 specimens of *Neobrachinus* and outgroups with bootstrap values. Phylogeny is color-coded by species group. Royal blue = polyphyletic fumans group. Red = misidentified specimens.

Table S1. Voucher specimens. Voucher number, collection information, and GenBank or BOLD accession numbers are provided for each specimen.

Voucher Species	Voucher Number	Location and Collecting Data	28S	CAD	COI
B. aabaaba<br>DNA5208	UASM365780	MEXICO: San Luis Potosi: 89km W of Ciudad del Maiz on Rte 80, 14 Oct 1965, coll. G.E. Ball, D.R. Whitehead			OP575849
B. adustipennis<br>DNA5207	UASM343307	MEXICO: Sonora: Mazatan, 13 Aug 2012, T.R. Van Devender, J. Palting	OP575866		
B. adustipennis<br>DNA2334		USA: AR: Montgomery Co., Lake Ouachita, 2 mi N Joplin off US 270, 11 Aug 2010, coll. Davidson, Acciavati, & Baldwin		KU937692	KU937768
B. adustipennis		USA: FL: Sarasota Co., Myakka River State Park			BBCCA508-12<br>BBCCA510-12<br>BBCCA522-12<br>BBCCA523-12<br>BBCCA524-12<br>BBCCA525-12
B. adustipennis	UAIC1012194	USA: GA: Bibb Co., Macon			NEONU719-11
B. aeger<br>DNA2335		NICARAGUA: Granada Prov., Domitila Reserva Silvestre Privada ESE Nandaime, 120m, 7 Jun 2005, coll. C.B. Barr, C.S. Chaboo	OP575867	KU937693	KU937769
B. alternans		USA: AL: Choctaw Co., Lenoir Landing NEON			BETN6017-19<br>BETN8994-20<br>BETN8975-20<br>BETN8995-20
B. alternans		USA: AL: Greene Co., Dead Lake NEON			BETN8912-20<br>BETN8992-20<br>BETN9009-20
B. alternans		USA: FL: Putnam Co., Ordway-Swisher Biological Station NEON			BETN2541-18
B. alternans		USA: KS: Douglas Co., KU Field Station NEON			BETN5784-19<br>BETN8589-20<br>BETN8625-20

					BETN8634-20<br>BETN8636-20
B. alternans<br>DNA5203	UASM268733	USA: KS: Douglas Co., State Lake, 17 July 1992, coll. B. Busby			OP575850
B. alternans		USA: KS: Jefferson Co., KU Field Station NEON			BETN8629-20<br>ISUP150-14<br>ISUP176-14<br>ISUP177-14<br>ISUP179-14<br>ISUP180-14<br>ISUP181-14<br>ISUP182-14<br>ISUP183-14<br>ISUP184-14<br>ISUP185-14<br>ISUP186-14
B. alternans		USA: KS: Riley Co., Konza Prairie Biological Station NEON			BETN8753-20<br>BETN8646-20<br>BETN5975-19
B. alternans		USA: MD: Anne Arundel Co., Smithsonian Environmental Research Center NEON			BETN1400-18<br>BETN4718-19
B. alternans<br>DNA2295		USA: TX: Potter Co., Plum Creek, 1 Oct 2008, coll. Vaughan, Jones	OP575868	KU937682	KU937757
B. americanus<br>DNA2311		USA: AR: Montgomery Co., Little Missouri Falls, 8 Apr 2011, coll. B. Baldwin	OP575870	KU937686	KU937762
B. americanus<br>DNA2355		USA: AR: Montgomery Co., Little Missouri Falls, 8 Apr 2011, coll. B. Baldwin	OP575869	KU937699	
B. armiger		Specific locality not reported	AF398693		
B. azureipennis<br>DNA2288	UAIC1047982	USA: AZ: Cochise Co., San Pedro River near Hwy. 82, 1180 m, 4 Oct 2012, coll. J. Schaller	OP575871	KU937676	KU937752
B. cibolensis<br>DNA5223	UASM348387	MEXICO: Sonora: Varela Ranch, Canon Bonita, 20-21 Aug 1982, coll. G.E. Ball, S. McCleve, D.R. Maddison			OP575851
B. cibolensis	UAIC1012200	USA: AZ: Cochise Co., Guadalupe Canyon			NEONU720-11

B. cinctipennis DNA5225	UASM367007	MEXICO: Jalisco: Lagos de Moreno, 7 Sep 1967, coll. T.L. Erwin, R.E. Leech		OP575852
B. cordicollis DNA2306		USA: PA: Lycoming Co. Loyalsock Creek, 4.8km E of Barbours, 230m, 7 May 2003, coll. M.J. Kowalski	KU937685	KU937761
B. costipennis	UAIC1012265	USA: AZ: Cochise Co., Guadalupe Canyon		NEONU725-11
B. costipennis DNA2181	UAIC1000016	USA: AZ: Cochise Co., San Pedro River, 1200m, 3 Jun 2004, coll. W. Moore	KU937643	KU937717
B. costipennis DNA2182	UAIC1000017	USA: AZ: Cochise Co., San Pedro River, 1200m, 3 Jun 2004, coll. W. Moore	KU937644	KU937718
B. costipennis	UAIC1012264	USA: AZ: Mohave Co., Burro Creek		NEONU723-11
B. costipennis DNA2274	UAIC1050075	USA: AZ: Pinal Co. 3 mi NE Winkleman, Gila River, 600m, 17 Mar 2011, coll. J. Schaller	KU937668	KU937744
B. costipennis		USA: CA: Los Angeles Co., Angeles National Forest		NEONU1838-13<br>NEONU1839-13
B. costipennis		USA: CA: Santa Barbara Co., Channel Islands National Park		NEONU1835-13<br>NEONU1836-13<br>NEONU1837-13
B. crepitans		CZECH REPUBLIC: Northern Bohemia: Rana u Loun, Oblik		KM450915<br>GBCOU1448-13
B. crepitans		GERMANY: Rhineland-Palatinate: Ahrweiler, Sinzig, Kiesgrube Goldene Meile		FBCOA712-10
B. crepitans		GERMANY: Rhineland-Palatinate: Alzey-Worms, Gundersheim, Weinberslage Hoellenbrand		FBCON015-13<br>GBCOG677-13
B. crepitans		GERMANY: Rhineland-Palatinate: Mayen-Koblenz, Plaidt, Gewerbegebiet		FBCOA944-10
B. crepitans		GERMANY: Rhineland-Palatinate: Sandgrube		GCOL3344-16<br>GCOL3345-16<br>GCOL3346-16<br>GCOL3347-16<br>GCOL3348-16
B. crepitans		GERMANY: Saxony-Anhalt, Schulmeisterberg sueld. Timmenrode		GCOL10013-16

B. crepitans		GERMANY: Saxony-Anhalt: Aseleben, Salziger See			FBCOJ789-13
B. crepitans		GERMANY: Saxony-Anhalt: Teufelsmauer bei Weddersleben			GCOL9804-16
B. crepitans DNA2206	UAIC1047973	GERMANY: Thuringia, Kullstedt, 7 May 2010	OP575872	KU937647	KU937721
B. crepitans		GERMANY: Thuringia: Rothenstein			GCOL1739-16
B. crepitans		GERMANY: Thuringia: Saxonyburg, Hainleite, Kammweg			GCOL3931-16<br>GCOL3932-16<br>GCOL3993-16
B. crepitans		SLOVAKIA: Specific locality not reported			JX412826
B. crepitans		SLOVENIA: Nova Gorica: Solkan, Sabotin			GBCOU3451-13<br>KM441050
B. cyanipennis DNA5214	UASM268773	CANADA: Ontario: Chaffeys Locks, 15 June 1985, coll. G.E. Ball			OP575853
B. cyanipennis		CANADA: Ontario: Hartington, Eel Lake Cottage,			MPCAN293-17<br>MPCAN294-17<br>MPCAN295-17
B. cyanochroaticus		USA: NC: Singetary Lake State Park, 21 May 2003, coll. H Cole			ICHC034-08
B. cyanochroaticus		USA: ND: Morton Co., Northern Great Plains Research Laboratory			BETN3212-19<br>BETN3330-19<br>BETN3331-19<br>BETN9268-20
B. cyanochroaticus		USA: ND: Stutsman Co., Chase Lake National Wildlife Refuge			BETN1736-18<br>BETN1747-18<br>BETN1755-18<br>BETN1778-18<br>BETN3266-19<br>BETN3267-19<br>BETN3268-19<br>BETN9110-20
B. elongatulus DNA5210	UASM373098	MEXICO: Sonora: Municipo de Fronteras, Rancho Capulin 23.5 km (by air) WSW Fronteras, Sierra Buenos Aires Ajos-Bavispe			OP575854

		Res., 1429m, 2 July 2016, T.R. Vandevender, J.D. Palting			
B. elongatulus		Specific locality not reported	KP419376		
B. elongatulus DNA2209	UAIC1000023	USA: AZ: Cochise Co. Chiricahua Mts., upper Turkey Creek, 2040m, 30-31 May 2010, coll. J. Schaller		KU937650	KU937722
B. elongatulus DNA2210	UAIC1000024	USA: AZ: Cochise Co. Chiricahua Mts., upper Turkey Creek, 2040m, 30-31 May 2010, coll. J. Schaller		KU937651	KU937723
B. elongatulus	UAIC1012364<br>UAIC1012365	USA: AZ: Cochise Co., Huachuca Mts., Parker Canyon			NEONU727-11<br>NEONU728-11
B. elongatulus		USA: AZ: Cochise Co., Santa Rita Mts. Box Canyon, 23 July 1994, coll. Olson			NEONU746-11
B. elongatulus DNA2278	UAIC1050512	USA: AZ: Maricopa Co., Lower Sycamore Creek, off Hwy, 720m, 28 Mar 2011, coll. J. Schaller		KU937672	KU937748
B. elongatulus DNA2271	UAIC1050511	USA: AZ: Pinal Co., 3 mi NE Winkleman, Gila River, 600m, 17 Mar 2011, coll. J. Schaller		KU937665	KU937741
B. elongatulus DNA2166	UAIC1000000	USA: AZ: Santa Cruz Co., Pajarito Mts., Sycamore Canyon, 1190m, 13 Mar 2010, coll. W.Moore, J. Schaller, R.L. Smith		KU937628	KU937704
B. elongatulus DNA2167	UAIC1000001	USA: AZ: Santa Cruz Co., Pajarito Mts., Sycamore Canyon, 1190m, 13 Mar 2010, coll. W.Moore, J. Schaller, R.L. Smith	OP575875	KU937629	KU937705
B. elongatulus DNA2168	UAIC1000002	USA: AZ: Santa Cruz Co., Pajarito Mts., Sycamore Canyon, 1190m, 13 Mar 2010, coll. W.Moore, J. Schaller, R.L. Smith	OP575874	KU937630	
B. elongatulus DNA2169	UAIC1000003	USA: AZ: Santa Cruz Co., Pajarito Mts., Sycamore Canyon, 1190m, 13 Mar 2010, coll. W.Moore, J. Schaller, R.L. Smith		KU937631	KU937703
B. elongatulus DNA2172	UAIC1000007	USA: AZ: Santa Cruz Co., Pajarito Mts., Sycamore Canyon, 1190m, 13 Mar 2010, coll. W.Moore, J. Schaller, R.L. Smith		KU937634	KU937708

B. elongatulus DNA2173	UAIC1000008	USA: AZ: Santa Cruz Co., Pajarito Mts., Sycamore Canyon, 1190m, 13 Mar 2010, coll. W.Moore, J. Schaller, R.L. Smith		KU937635	KU937709
B. elongatulus DNA2174	UAIC1000009	USA: AZ: Santa Cruz Co., Pajarito Mts., Sycamore Canyon, 1190m, 13 Mar 2010, coll. W.Moore, J. Schaller, R.L. Smith		KU937636	KU937710
B. elongatulus DNA2226	UAIC1000029	USA: AZ: Santa Cruz Co., Pajarito Mts., Sycamore Canyon, 1190m, 13 Mar 2010, coll. W.Moore, J. Schaller, R.L. Smith	OP575873	KU937656	KU937728
B. elongatulus DNA2227	UAIC1000030	USA: AZ: Santa Cruz Co., Pajarito Mts., Sycamore Canyon, 1190m, 13 Mar 2010, coll. W.Moore, J. Schaller, R.L. Smith		KU937657	KU937729
B. elongatulus	UAIC1012309	USA: AZ: Santa Cruz Co., Rock Corral Canyon Tumacacori Mts.			NEONU726-11
B. elongatulus		USA: OK: Marshall Co., Oklahoma University Biological Station			OUENT091-09
B. elongatulus		USA: TX: Comal Co., Bulverde, 20km NNE San Antonio			USCOL191-09<br>USCOL192-09<br>USCOL193-09<br>USCOL194-09
B. elongatulus		USA: TX: Palo Duro Canyon State Park			BBCCA515-12<br>BBCCA516-12<br>BBCCA518-12<br>BBCCA519-12<br>BBCCA520-12<br>BBCCA526-12<br>BBCCA527-12<br>BBCCA3465-12
B. elongatulus		USA: TX: Sutton Co., Sonora Caverns, 15 km W of Sonora, 680m, 22-23 May 2009, coll. K. Perez, S. McCubbin, J. Cossey, A. Borisenko			USCOL195-09<br>USCOL196-09<br>USCOL197-09
B. explodens		GERMANY: Baden-Wuerttemberg: Ihringen, Winklerberg, 250m, 25 Aug 2013, coll. GBOL-Team ZFMK			GCOL7759-16<br>GCOL7760-16

B. explodens		GERMANY: Baden-Wuerttemberg: Vogtsburg-Altvogtsburg, Haselschacher Buck			FBCOK014-13
B. explodens		GERMANY: Rhineland-Palatinate: Bad Duerkheim, Freinsheim, Sastanienhain Im Hahnen			GBCOF186-13
B. explodens		GERMANY: Rhineland-Palatinate: Gundersheim, Germersheim, Jockgrim, Sandmagerrasen			KM442145<br>KM446858<br>FBCOP901-13<br>FBCOP902-13<br>GBCOL768-12<br>GBCOL769-12
B. explodens		GERMANY: Rhineland-Palatinate: Gundersheim, Weinbergslage Hoellenbrand			FBCON055-13<br>GBCOG712-13<br>GBCOG713-13
B. explodens		GERMANY: Saxony-Anhalt: Halberstadt, Lkr. Harz, westl. Athenstedt			GCOL9273-16
B. explodens		GERMANY: Thuringia: Bad Blankenburg, Boehlscheiben			GCOL4117-16
B. explodens		GERMANY: Thuringia: Jena, Cospoth			GCOL6034-16
B. explodens		GERMANY: Thuringia: Oberheldrungen, Acker noerdlich			GCOL6041-16
B. explodens		SLOVENIA: Gorenjska: Poljane Sora Valley, Hotavlje			FBCOK533-13
B. explodens		SLOVENIA: Nova Gorica, Osek, Vogrscek			GBCOU3439-13
B. explodens		SLOVENIA: Severnoprimorska: Solkan, Sabotin			GCOL7761-16<br>GCOL7762-16
B. favicollis DNA2279	UAIC1050056	USA: AZ: Maricopa Co., Lower Sycamore Creek, off Hwy, 720m 28 Mar 2011, coll. J. Schaller		KU937673	KU937749
B. favicollis DNA2207	UAIC1000021	USA: AZ: Yavapai Co., T18N, R6W sec. 25, Walnut Creek, 8 Jul 2004, coll. R. Delph	OP575876	KU937648	
B. favicollis DNA2208	UAIC1000022	USA: AZ: Yavapai Co., T18N, R6W sec. 25, Walnut Creek, 8 Jul 2004, coll. R. Delph	OP575878	KU937649	

B. favicollis DNA2272	UAIC1050055	USA: AZ: Pinal Co., 3 mi NE Winkleman, Gila River, 600m, 17 Mar 2011, coll. J. Schaller	OP575877	KU937666	KU937742
B. favicollis		USA: CA: Los Angeles Co., Angeles National Forest: SDEF			NEONU1832-13<br>NEONU1834-13
B. favicollis		USA: CA: Santa Barbara Co., Los Padres National Forest: Oso Canyon			NEONU1833-13
B. fulminatus DNA2264		USA: MA: Harvard Forest Site, 340m, 5 July 2010, coll. NEON	OP575879	KU937662	KU937738<br>NEONT400-10
B. fumans DNA2289	UAIC1047987	MEXICO: Sonora, La Colorada, E side of Tecoripa, 410m, 22 Sep 2011, coll. T.R. VanDevender, A.L. Reina-G.	OP575882	KU937677	KU937753
B. fumans DNA2290	UAIC1047988	MEXICO: Sonora, Municipio de Carbo. El Oasis on MEX 15, 7.9km W Carbo, 550m, 16 Oct 2011, coll. T.R. VanDevender.	OP575881	KU937678	KU937754
B. fumans DNA5209	UASM370731	MEXICO: Sonora, Municipio de Agua Prieta, 8 Aug 2015, coll. T.R. Van Devender, A.L. Reina-G	OP575880		OP575855
B. fumans		USA: MD: Anne Arundel Co., Smithsonian Environmental Research Center			BETN4788-19<br>BETN7863-20<br>BETN7867-20<br>BETN7868-20
B. fumans		USA: NM: Otero Co., White Sands National Monument			NEONU1127-12<br>NEONU1128-12
B. fumans		USA: VA: Clarke Co., Blandy Experimental Farm			BETN1369-18<br>BETN5015-19<br>BETN7864-20<br>BETN7865-20
B. fumans		USA: VA: Warren Co., Smithsonian Conservation Biology Institute			BETN4852-19<br>BETN4853-19<br>BETN7866-20<br>BETN7869-20<br>BETN7870-20
B. galactoderus DNA5216	UASM367811	MEXICO: Guerrero: 66.6km N Acapulco, 20 Dec 1965, coll. G.E. Ball, D.R. Whitehead			OP575856

B. galactoderus DNA5217	UASM367812	MEXICO: Sinaloa: Rio Panuco, 18.0km NE Concordia on Rte 40, 12 Jan 1966, coll. D.R. Whitehead			OP575857
B. gebhardis DNA5211	UASM370729	MEXICO: Sonora: Pilares de Nacozari, 8 Aug 2015, coll. D. Shpeley	OP575885		OP575858
B. gebhardis DNA2253	UAIC1050005	USA: AZ: Pima Co., Tanque Verde Creek, trail off Reddington Rd., 860m, 27 May 2010, coll. J. Schaller	OP575883		KU937734
B. gebhardis DNA2254	UAIC1050006	USA: AZ: Catalina Mountains, 2013, coll. J. Evle.			2254
B. gebhardis DNA2280	UAIC1050007	USA: AZ: Maricopa Co. Lower Sycamore Creek, off Hwy. 87, 720m 28 Mar 2011, coll. J. Schaller	OP575884	KU937674	KU937750
B. gebhardis		USA: CA: Los Angeles Co., Angeles National Forest: Ruby Canyon			NEONU1829-13
B. gebhardis		USA: CA: Santa Barbara Co., Channel Islands National Park: Santa Rosa Island			NEONU1827-13 NEONU1828-13
B. gebhardis		USA: CA: Santa Barbara Co., Los Padres National Forest: Birbent Canyon			NEONU1831-13
B. gebhardis		USA: CA: Santa Barbara Co., UC Sedgwick Reserve			NEONU1830-13
B. geniculatus DNA2300		PANAMA: Rio Manoni, 1 Sep 2006	OP575886	KU937683	KU937759
B. hirsutus		Specific locality not reported	AF398693		
B. hirsutus DNA2179	UAIC1000014	USA: AZ: Cochise Co., San Pedro River, Charleston Bridge, 1200m, 23 Feb 2010, coll. W.Moore, D. and J. Maddison	OP575889	KU937641	KU937715
B. hirsutus	UAIC1012751	USA: AZ: Graham Co., Bonita Creek			NEONU752-11
B. hirsutus DNA2273	UAIC1050158	USA: AZ: Pinal Co. 3 mi NE Winkleman, Gila River, 600m, 17 Mar 2011, coll. J. Schaller	OP575887	KU937667	KU937743
B. hirsutus DNA2275	UAIC1050159	USA: AZ: Maricopa Co. Lower Sycamore Creek, 720m 28 Mar 2011, coll. J. Schaller	OP575888	KU937669	KU937745

B. hirsutus DNA2262	UAIC1050162	USA: AZ: Santa Cruz Co. Atascosa Mts. Pena Blanca Lake, 1170m, 7 Nov 2010 J. Schaller	OP575890		KU937736
B. hirsutus	UAIC1012764<br>UAIC1012866	USA: AZ: Mohave Co., 6 mi W of signal Big Sandy River			NEONU753-11<br>NEONU755-11
B. hirsutus	UAIC1012765	USA: AZ: Mohave Co., 6.9 mi NE of Nothing			NEONU754-11
B. hirsutus DNA2180	UAIC1000015	USA: AZ: Santa Cruz Co., Pajarito Mts., Sycamore Canyon, 1190m, 13 Mar 2010, coll. W.Moore, J. Schaller, R.L. Smith		KU937642	KU937716
B. hirsutus		USA: AZ: Yavapai Co., Willow Creek Reservoir, 3 km NE Prescott			USCOL188-09
B. imperialensis		USA: KS: Douglas Co., KU Field Station			BETN337-18<br>BETN338-18
B. imperialensis		USA: KS: Jefferson Co., KU Field Station			BETN8592-20<br>BETN8609-20
B. imporcitis DNA2277	UAIC1050025	USA: AZ, Maricopa Co. Lower Sycamore Creek, 720m, 28 Mar 2011, coll. J. Schaller	OP575891	KU937671	KU937747
B. janthinipennis		CANADA: Ontario: Rouge National Urban Park			RBINA5443-13
B. janthinipennis DNA5229		USA: KS: Sedgwick Co., Ninnescah Biological Reserve, 2 Sep 2017, coll. E. Waite	OP575893		OP575860
B. janthinipennis DNA5205	UASM268815	USA: MA: Nantucket Co., Nantucket Island, Sesachacha Pond, 3 Sep 1995, coll. F. Purrington	OP575892		OP575859
B. janthinipennis DNA2323		USA: OK: Custer Co. Foss State Park, Sandy Beach, 06 Oct 2012, coll. G.F. Hevel	OP575894	KU937691	KU937767
B. javalinopsis DNA2287	UAIC1003101	USA: AZ: Cochise Co., San Pedro River near Hwy. 82, 1180 m, 1 Jun 2011, coll. J. Schaller		KU937675	KU937751
B. javalinopsis	UAIC1012877	USA: AZ: Cochise Co., Texas Canyon L. Dragoon Mts.			NEONU758-11
B. javalinopsis		USA: NM: Otero Co., White Sands National Monument			NEONU1129-12<br>NEONU1130-12

					NEONU1131-12<br>NEONU1132-12
B. kavanaughi		USA: AZ: Yavapai Co., Willow Creek Reservoir, 3 km NE Prescott			USCOL189-09<br>USCOL190-09
B. kavanaughi		USA: CA: Point Mugu State Park			BBCCA521-12
B. kavanaughi	UAIC1012886	USA: TX: Blanco Co., Federnales Falls State Park			NEONU736-11
B. lateralis<br>DNA2183	UAIC1000018	USA: AZ, Santa Cruz Co., Patagonia Lake State Park, 1150m, 6 Jun 2004, coll. J.C. Oliver and K.L. Prudic	OP575895	KU937645	KU937719
B. lateralis<br>DNA2270	UAIC1047998	USA: AZ, Pinal Co. 3 mi NE Winkleman, Gila River, 600m, 17 Mar 2011, coll. J. Schaller	OP575896	KU937664	KU937740
B. lateralis<br>DNA2261	UAIC1047999	USA: AZ: Santa Cruz Co. Atascosa Mts. Pena Blanca Lake, 1170m, 7 Nov 2010 J. Schaller	OP575897		KU937735
B. medius		USA: FL: Sarasota Co., Myakka River State Park			BBCCA535-12
B. medius		USA: ND: Morton Co., Northern Great Plains Research Laboratory			BETN1810-18<br>BETN9260-20
B. medius		USA: ND: Stutsman Co., Chase Lake National Wildlife Refuge			BETN1837-18<br>BETN9117-20<br>BETN9121-20
B. medius		USA: TX: Palo Duro Canyon State Park			BBCCA533-12<br>BBCCA534-12
B. mexicanus<br>DNA2178	UAIC1000013	USA: AZ: Cochise Co., San Pedro River, Charleston Bridge, 1200m, 23 Feb 2010, coll. W.Moore, D. and J. Maddison		KU937640	KU937714
B. mexicanus<br>DNA2276	UAIC1050347	USA: AZ: Maricopa Co., Lower Sycamore Creek, off Hwy, 720m, 28 Mar 2011, coll. J. Schaller		KU937670	KU937746
B. mexicanus<br>DNA2269	UAIC1050348	USA: AZ: Gila Co., 3 mi NE Winkleman, Gila River, 600m, 17 Mar 2011, coll. J. Schaller	OP575899	KU937663	KU937739

B. mexicanus	UAIC1011761<br>UAIC1012916	USA: AZ: Pinal Co., Buehman Creek near Reddington			NEONU741-11<br>NEONU742-11
B. mexicanus<br>DNA2263	UAIC1050350	USA: AZ: Santa Cruz Co., Atascosa Mts. Pena Blanca Lake, 1170m, 7 Nov 2010 J. Schaller	OP575900		KU937737
B. mexicanus<br>DNA2170	UAIC1000004	USA: AZ: Santa Cruz Co., Pajarito Mts., Sycamore Canyon, 1190m, 13 Mar 2010, coll. W. Moore, J. Schaller, R.L. Smith		KU937632	KU937706
B. mexicanus<br>DNA2171	UAIC1000005	USA: AZ: Santa Cruz Co., Pajarito Mts., Sycamore Canyon, 1190m, 13 Mar 2010, coll. W. Moore, J. Schaller, R.L. Smith		KU937633	KU937707
B. mexicanus<br>DNA2177	UAIC1000012	USA: AZ: Santa Cruz Co., Pajarito Mts., Sycamore Canyon, 1190m, 13 Mar 2010, coll. W. Moore, J. Schaller, R.L. Smith		KU937639	KU937713
B. mexicanus<br>DNA2223	UAIC1000026	USA: AZ: Santa Cruz Co., Pajarito Mts., Sycamore Canyon, 1190m, 13 Mar 2010, coll. W. Moore, J. Schaller, R.L. Smith	OP575902	KU937653	KU937725
B. mexicanus<br>DNA2224	UAIC1000027	USA: AZ: Santa Cruz Co., Pajarito Mts., Sycamore Canyon, 1190m, 13 Mar 2010, coll. W. Moore, J. Schaller, R.L. Smith	OP575898	KU937654	KU937726
B. mexicanus<br>DNA2225	UAIC1000028	USA: AZ: Santa Cruz Co., Pajarito Mts., Sycamore Canyon, 1190m, 13 Mar 2010, coll. W. Moore, J. Schaller, R.L. Smith	OP575901	KU937655	KU937727
B. mexicanus<br>DNA2205	UAIC1000019	USA: AZ: Yavapai Co.,T18N, R6W sec. 25, Walnut Creek, 8 Jul 2004, coll. R. Delph	OP575903	KU937646	
B. neglectus<br>DNA2316		USA: SC: Hampton Co., 7km WSW Garnett, Webb Wildlife Mgt. Area, Bluff Lake, 19m, 7 Apr 2006, coll. R. Androw, K. Karns	OP575904	KU937687	KU937763
B. ovipennis<br>DNA5230		USA: PA: Lehigh Co., Trexler Nature Preserve, 20 Nov 2017, coll. E. Waite	OP575905		OP575861
B. ovipennis<br>DNA2222	UAIC1047981	USA: VT: Chittenden Co., Colchester, Delta Park, 30m, 15 Jun 2010, coll. W. Moore		KU937652	KU937724
B. pallidus<br>DNA2299		USA: CA: Yolo Co. Rd. 57, Cache Creek, 130m	OP575906	KU937680	KU937758
B. perplexus		USA: AL: Greene Co., Dead Lake			BETN715-18

B. perplexus		USA: CO: Kiowa Co., Off Colorado Road 54			NEONU005-11
B. phaeocerus DNA2175	UAIC1000010	USA: AZ: Cochise Co., San Pedro River, Charleston Bridge, 1200m, 23 Feb 2010, coll. W. Moore, D. and J. Maddison	OP575909	KU937637	KU937711
B. phaeocerus DNA2176	UAIC1000011	USA: AZ: Cochise Co., San Pedro River near Hwy 82, 1180m, 1 June 2011, coll. J. Schaller	OP575910	KU937638	KU937712
B. phaeocerus		USA: CO: Kiowa Co., Off Colorado Road 54			NEONU021-11
B. phaeocerus DNA2321		USA: OK: Washita Co., Rocky, Rocky Lake, 7 Oct 2012, coll. G.F. Hevel	OP575908	KU937689	KU937765
B. phaeocerus DNA2322		USA: OK: Washita Co., Rocky, Rocky Lake, 7 Oct 2012, coll. G.F. Hevel	OP575907	KU937690	KU937766
B. quadripennis DNA2336	UAIC1047978	USA: FL: Columbia Co. Osceola NF, 40m, 18 Aug 2012, coll. P. Gorring, L. Davis	OP575911	KU937694	KU937770
B. quadripennis DNA2337	UAIC1047977	USA: FL: Columbia Co. Osceola NF, 40m, 18 Aug 2012, coll. P. Gorring, L. Davis	OP575912	KU937695	KU937702
B. quadripennis DNA5212	UASM268747	USA: FL: Highlands Co., Archbold Biological Station, 20 Apr 2002, coll. V. Golia			OP575862
B. quadripennis		USA: FL: Highlands Co., Archbold Biological Station			BBCCA509-12
B. quadripennis		USA: FL: Kissimmee Prairie Preserve SP			BBCCA511-12
B. quadripennis		USA: FL: Kissimmee Prairie Preserve SP, Hammock Trail			BBCCA513-12 BBCCA514-12
B. quadripennis		USA: FL: Sarasota Co., Myakka River State Park			BBCCA507-12 BBCCA517-12
B. quadripennis		USA: GA: Savannah Co.			BBCCA512-12
B. sclopeta		PORTUGAL: Braganca: Mirandela, Romeu			OSOIL003-16
B. scopleta DNA2320		ITALY: Sicily, Palermo Prov., Piano Battaglia, Parc Regione delle Madonie, 1560m, 11 May 2011, D. Brzoska		KU937688	KU937764
B. sublaevis DNA2356		USA: AR: Garland Co., FS Camp Clearfork, 20 Jun 2009, coll. B. Baldwin	OP575913	KU937700	

B. tenuicollis		USA: AK: Toad Suck Park			BBCCA501-12
B. (Brachynolomus) sp. J DNA2339	UAIC1047975	ITALY: Lazio: Acropoli di Tarquinia (vr), 10 Mar 2013, coll. M. Bologna		KU937697	
B.(Brachynolomus) sp.K DNA2340	UAIC1047976	ITALY: Lazio: Acropoli di Tarquinia (vr), 10 Mar 2013, coll. M. Bologna		KU937698	
B. (Metabrachinus) sp. A DNA2292	UAIC1047966	TANZANIA: Morogoro, Mvomelo District, Milama, 13 Nov. 2011, coll. K. Angelus		KU937679	KU937755
B. (Metabrachinus) sp. B DNA2294	UAIC1047967	TANZANIA: Morogoro, Mvomelo District, Milama, 13 Nov. 2011, coll. K. Angelus	OP575863	KU937681	KU937756
B. (Metabrachinus) sp. D DNA2234	UAIC1047964	SOUTH AFRICA: Limpopo, Waterberg Mts.,1490m, 5 Dec 2003, coll. W. Moore	OP575864	KU937661	KU937733
B. (Metabrachinus) sp. D DNA2233	UAIC1047963	SOUTH AFRICA: Mpumalanga, Berlin Forest, 920m, 14 Dec 2003, coll. W. Moore	OP575865	KU937660	KU937732
B. (Neobrachinus) sp. C DNA2303		PARAGUAY: Central Department Capiata, 7 Dec 2005, coll. K. Will		KU937684	KU937760
B. (Neobrachinus) sp. E DNA2318		GUYANA: Region 9, Karanambu Lodge, 14-17 Sep 2012, 100m, coll. D. Brzoska			KU937701
